# Genetic tools weed out misconceptions of strain reliability in *Cannabis sativa:* implications for a budding industry

**DOI:** 10.1186/s42238-019-0001-1

**Published:** 2019-06-07

**Authors:** Anna L. Schwabe, Mitchell E. McGlaughlin

**Affiliations:** School of Biological Sciences, University of Northern Colorado, Greeley, CO USA

**Keywords:** *Cannabis*, *Cannabis sativa*, Consumer, Genotype, Hemp, Marijuana, Microsatellite, Phenotype, Strain

## Abstract

**Background:**

Unlike other plants, *Cannabis sativa* is excluded from regulation by the United States Department of Agriculture (USDA). Distinctive *Cannabis* varieties are ostracized from registration and therefore nearly impossible to verify. As *Cannabis* has become legal for medical and recreational consumption in many states, consumers have been exposed to a wave of novel *Cannabis* products with many distinctive names. Despite more than 2000 named strains being available to consumers, questions about the consistency of commercially available strains have not been investigated through scientific methodologies. As *Cannabis* legalization and consumption increases, the need to provide consumers with consistent products becomes more pressing. In this research, we examined commercially available, drug-type *Cannabis* strains using genetic methods to determine if the commonly referenced distinctions are supported and if samples with the same strain name are consistent when obtained from different facilities.

**Methods:**

We developed ten *de-novo* microsatellite markers using the “Purple Kush” genome to investigate potential genetic variation within 30 strains obtained from dispensaries in three states. Samples were examined to determine if there is any genetic distinction separating the commonly referenced Sativa, Indica and Hybrid types and if there is consistent genetic identity found within strain accessions obtained from different facilities.

**Results:**

Although there was strong statistical support dividing the samples into two genetic groups, the groups did not correspond to commonly reported Sativa/Hybrid/Indica types. The analyses revealed genetic inconsistencies within strains, with most strains containing at least one genetic outlier. However, after the removal of obvious outliers, many strains showed considerable genetic stability.

**Conclusions:**

We failed to find clear genetic support for commonly referenced Sativa, Indica and Hybrid types as described in online databases. Significant genetic differences within samples of the same strain were observed indicating that consumers could be provided inconsistent products. These differences have the potential to lead to phenotypic differences and unexpected effects, which could be surprising for the recreational user, but have more serious implications for patients relying on strains that alleviate specific medical symptoms.

**Electronic supplementary material:**

The online version of this article (10.1186/s42238-019-0001-1) contains supplementary material, which is available to authorized users.

## Background

Cultivation of *Cannabis sativa* L. dates back thousands of years (Abel 2013) but has been largely illegal worldwide for the best part of the last century. The U.S. Drug Enforcement Agency considers *Cannabis* a Schedule I drug with no “accepted medical use in treatment in the United States” (United States Congress n.d.), but laws allowing *Cannabis* for use as hemp, medicine, and some adult recreational use are emerging (ProCon 2018). Global restrictions have limited *Cannabis* related research, and there are relatively few genetic studies focused on strains (Lynch et al. 2016; Soler et al. 2017), but studies with multiple accessions of a particular strain show variation (Lynch et al. 2016; Soler et al. 2017; Sawler et al. 2015).

Currently, the *Cannabis* industry has no way to verify strains. Consequently, suppliers are unable to provide confirmation of strains, and consumers have to trust the printed name on a label matches the product inside the package. Reports of inconsistencies, along with the history of underground trading and growing in the absence of a verification system, reinforce the likelihood that strain names may be unreliable identifiers for *Cannabis* products at the present time. Without verification systems in place, there is the potential for misidentification and mislabeling of plants, creating names for plants of unknown origin, and even re-naming or re-labeling plants with prominent names for better sale. *Cannabis* taxonomy is complex (Emboden 1974; Schultes et al. 1974; Hillig 2005; Russo 2007; Clarke and Merlin 2013; Clarke et al. 2015; Clarke and Merlin 2016; Small et al. 1976; Small 2015a), but given the success of using genetic markers, such as microsatellites, to determine varieties in other crops, we suggest that similar genetic based approaches should be used to identify *Cannabis* strains in medical and recreational marketplaces.

There are an estimated ~ 3.5 million medical marijuana patients in the United States (U.S.) (Leafly 2018b) and various levels of recent legalization in many states has led to a surge of new strains (Leafly 2018a; Wikileaf 2018). Breeders are producing new *Cannabis* strains with novel chemical profiles resulting in various psychotropic effects and relief for an array of symptoms associated with medical conditions including (but not limited to): glaucoma (Tomida et al. 2004), Chron’s Disease (Naftali et al. 2013), epilepsy (U.S. Food and Drug Administration 2018; Maa and Figi 2014), chronic pain, depression, anxiety, PTSD, autism, and fibromyalgia (Naftali et al. 2013; Cousijn et al. 2018; Ogborne et al. 2000; Borgelt et al. 2013; ProCon 2016).

There are primarily two *Cannabis* usage groups, which are well supported by genetic analyses (Lynch et al. 2016; Soler et al. 2017; Sawler et al. 2015; Dufresnes et al. 2017): ***hemp*** defined by a limit of < 0.3% Δ^9^-tetrahydrocannabinol (THC) in the U.S., and ***marijuana*** or ***drug-types*** with moderate to high THC concentrations (always > 0.3% THC). Within the two major groups *Cannabis* has been further divided into strains (varietals) in the commercial marketplace, and particularly for the drug types, strains are assigned to one of three categories: ***Sativa*** which reportedly has uplifting and more psychotropic effects, ***Indica*** which reportedly has more relaxing and sedative effects, and ***Hybrid*** which is the result of breeding Sativa and Indica types resulting in intermediate effects. The colloquial terms **Sativa, Hybrid**, and **Indica** are used throughout this document even though these terms do not align with the current formal botanical taxonomy for *Cannabis sativa* and proposed *Cannabis indica* (McPartland 2017; Piomelli and Russo 2016). We feel the colloquial terminology is necessary here as the approach for this study was from a consumer view, and these are the terms offered as common descriptors for the general public (Leafly 2018a; Wikileaf 2018; cannabis.info 2018; NCSM 2018; PotGuide.com 2018; Seedfinder 2018). Genetic analyses have not provided a clear consensus for higher taxonomic distinction among these commonly described *Cannabis* types (Lynch et al. 2016; Sawler et al. 2015), and whether there is a verifiable difference between Sativa and Indica type strains is debated (McPartland 2017; Piomelli and Russo 2016; Erkelens and Hazekamp 2014). However, both the recreational and medical *Cannabis* communities claim there are distinct differences in effects between Sativa and Indica type strains (Leafly 2018a; Wikileaf 2018; cannabis.info 2018; NCSM 2018; PotGuide.com 2018; Seedfinder 2018; Leaf Science 2016; Smith 2012).

Female *Cannabis* plants are selected based on desirable characters (mother plants) and are produced through cloning and, in some cases, self-fertilization to produce seeds (Green 2005). Cloning allows *Cannabis* growers to replicate plants, ideally producing consistent products. There are an overwhelming number of *Cannabis* strains that vary widely in appearance, taste, smell and psychotropic effects (Leafly 2018a; Wikileaf 2018; cannabis.info 2018; NCSM 2018; PotGuide.com 2018; Seedfinder 2018). Online databases such as Leafly (2018a) and Wikileaf (2018), for example, provide consumers with information about strains but lack scientific merit for the *Cannabis* industry to regulate the consistency of strains. Other databases exist (cannabis.info 2018; NCSM 2018; PotGuide.com 2018; Seedfinder 2018), but the method of assignment to the three groups is often undisclosed, confounded, or mysterious. Wikileaf reports a numeric percentage of assignment to Sativa and/or Indica (Wikileaf 2018), which is why we chose it as our reference scale of ancestry, although there is some disagreement among online sources (Additional file 1: Table S1). To our knowledge, there have not been any published scientific studies specifically investigating the genetic consistency of strains at multiple points of sale for *Cannabis* consumers.

Breeders and growers choose *Cannabis* plants with desirable characters (phenotype) related to flowers, cannabinoid profile, and terpene production. Phenotype is a product of genotype and environment. *Cannabis* is considerably variable and extraordinarily plastic in response to varying environmental conditions (Onofri and Mandolino 2017). Therefore, determining sources of variation, at the most basic level, requires examining genetic differences. Strains propagated through cloning should have minimal genetic variation. Eight of the strains examined in this study are reportedly clone only strains indicating there should be little to no genetic variation within these strains. That being said, it is possible for mutations to accumulate over multiple generations of cloning (Gabriel et al. 1993; Hojsgaard and Horandl 2015), but these should not be widespread. Self-fertilization and subsequent seed production may also be used to grow a particular strain. With most commercial plant products growers go through multiple generations of self-fertilization and backcrossing to remove genetic variability within a strain and provide a consistent product (Riggs 1988). However, for many *Cannabis* strains, the extent of genetic variability stabilization is uncertain. It has been observed that novel *Cannabis* strains developed through crossing are often phenotypically variable (Green 2005), which could be the result of seed producers growing seeds that are not stabilized enough to produce a consistent phenotype. Soler et al. (2017) examined the genetic diversity and structure of *Cannabis* cultivars grown from seed and found considerable variation, suggesting that seed lots are not consistent. Given the uncertainties surrounding named *Cannabis* strains, genetic data provides an ideal path to examine how widespread genetic inconsistencies might be.

In the U.S., protection against commercial exploitation, trademarking, and recognition of intellectual property for developers of new plant cultivars is provided through the United States Department of Agriculture (USDA) and The Plant Variety Protection Act of 1970 (United States Department of Agriculture 1970). Traditionally, morphological characters were used to define new varieties in crops such as grapes (*Vitis vinifera* L.), olives (*Olea europea* L.) and apples (*Malus domestica* Borkh.). With the rapid development of new varieties in these types of crops, morphological characters have become increasingly difficult to distinguish. Currently, quantitative and/or molecular characters are often used to demonstrate uniqueness among varieties. Microsatellite genotyping enables growers and breeders of new cultivars to demonstrate uniqueness through variable genetic profiles (Rongwen et al. 1995). Microsatellite genotyping has been used to distinguish cultivars and hybrid varieties of multiple crop varietals within species (Rongwen et al. 1995; Guilford et al. 1997; Hokanson et al. 1998; Cipriani et al. 2002; Belaj et al. 2004; Sarri et al. 2006; Baldoni et al. 2009; Stajner et al. 2011; Costantini et al. 2005; Pellerone et al. 2001; Poljuha et al. 2008; Muzzalupo et al. 2009). Generally, 3–12 microsatellite loci are sufficient to accurately identify varietals and detect misidentified individuals (Cipriani et al. 2002; Belaj et al. 2004; Sarri et al. 2006; Baldoni et al. 2009; Poljuha et al. 2008; Muzzalupo et al. 2009). *Cannabis* varieties however, are not afforded any legal protections, as the USDA considers it an “ineligible commodity” (United States Department of Agriculture 2014) but genetic variety identification systems provide a model by which *Cannabis* strains could be developed, identified, registered, and protected.

We used a well-established genetic technique to compare commercially available *C. sativa* strains to determine if products with the same name purchased from different sources have genetic congruence. This study is highly unique in that we approached sample acquisition as a common retail consumer by purchasing flower samples from dispensaries based on what was available at the time of purchase. All strains were purchased as-is, with no additional information provided by the facility, other than the identifying label. This study aimed to determine if: (1) any genetic distinction separates the common perception of Sativa, Indica and Hybrid types; (2) consistent genetic identity is found within a variety of different strain accessions obtained from different facilities; (3) there is evidence of misidentification or mislabeling.

## Methods

### Genetic material

*Cannabis* samples for 30 strains were acquired from 20 dispensaries or donors in three states (Table 1). All samples used in this study were obtained legally from either retail (Colorado and Washington), medical (California) dispensaries, or as a donation from legally obtained samples (Greeley 1). DNA was extracted using a modified CTAB extraction protocol (Doyle 1987) with 0.035–0.100 g of dried flower tissue per extraction. Several databases exist with various descriptive Sativa and Indica assignments for thousands of strains (Additional file 1: Table S1). For this study proportions of Sativa and Indica phenotypes from Wikileaf (2018) were used. Analyses were performed on the full 122-sample dataset (Table 1). The 30 strains were assigned a proportion of Sativa according to online information (Table 2). Twelve of the 30 strains were designated as ‘popular’ due to higher availability among the dispensaries as well as online information reporting the most popular strains (Table 2) (Rahn 2015; Rahn 2016; Rahn et al. 2016; Escondido 2014). Results from popular strains are highlighted to show levels of variation in strains that are more widely available or that are in higher demand.

**Table 1:** *Cannabis* samples (122) from 30 strains with the reported proportion of Sativa from Wikileaf (2018) and the city location and state where each sample was acquired. (SLO: San Luis Obispo)

Name	Sativa	City	State	Name	Sativa	City	State
Durban Poison	100	Boulder 1	CO	OG Kush	55	Denver 3	CO
Durban Poison	100	Boulder 3	CO	OG Kush	55	Fort Collins 3	CO
Durban Poison	100	Denver 1	CO	OG Kush	55	Garden City 2	CO
Durban Poison	100	Denver 2	CO	OG Kush	55	SLO 1	CA
Durban Poison	100	Fort Collins 3	CO	Blue Dream	50	Boulder 1	CO
Durban Poison	100	Fort Collins 4	CO	Blue Dream	50	Boulder 2	CO
Durban Poison	100	Garden City 1	CO	Blue Dream	50	Boulder 3	CO
Durban Poison	100	Garden City 2	CO	Blue Dream	50	Denver 1	CO
Durban Poison	100	Union Gap 1	WA	Blue Dream	50	Garden City 4	CO
Hawaiian	90	Boulder 1	CO	Blue Dream	50	Garden City 4	CO
Hawaiian	90	Fort Collins 2	CO	Blue Dream	50	SLO 2	CA
Sour Diesel	90	Boulder 1	CO	Blue Dream	50	SLO 3	CA
Sour Diesel	90	Boulder 3	CO	Blue Dream	50	SLO 4	CA
Sour Diesel	90	Greeley 1	CO	Tahoe OG	50	Boulder 1	CO
Sour Diesel	90	Denver 4	CO	Tahoe OG	50	Denver 1	CO
Sour Diesel	90	Fort Collins 3	CO	Tahoe OG	50	Fort Collins 4	CO
Sour Diesel	90	Garden City 1	CO	Tahoe OG	50	SLO 3	CA
Sour Diesel	90	Garden City 2	CO	ChemdawgD^a^	40	Boulder 1	CO
Trainwreck	90	Denver 1	CO	ChemDawg	45	Boulder 2	CO
Trainwreck	90	Garden City 1	CO	ChemDawg	45	Boulder 3	CO
Island Sweet Skunk	80	Boulder 1	CO	ChemdawgD^a^	40	Denver 1	CO
Island Sweet Skunk	80	Garden City 1	CO	Chemdawg 91	40	Denver 5	CO
Island Sweet Skunk	80	Garden City 2	CO	Chemdog 1^a^	40	Garden City 1	CO
AK-47	65	Boulder 1	CO	ChemDawg	45	Garden City 2	CO
AK-47	65	Denver 3	CO	Headband	45	Garden City 1	CO
AK-47	65	SLO 2	CA	Headband	45	Greeley 1	CO
Golden Goat	65	Boulder 1	CO	Banana Kush	40	Denver 1	CO
Golden Goat	65	Boulder 2	CO	Banana Kush	40	Garden City 1	CO
Golden Goat	65	Boulder 3	CO	Banana Kush	40	Garden City 2	CO
Golden Goat	65	Denver 1	CO	Banana Kush	40	Greeley 1	CO
Golden Goat	65	Garden City 1	CO	Girl Scout Cookies	40	Boulder 1	CO
Golden Goat	65	Garden City 1	CO	Girl Scout Cookies	40	Denver 1	CO
Golden Goat	65	Garden City 2	CO	Girl Scout Cookies	40	Fort Collins 2	CO
Green Crack	65	Fort Collins 2	CO	Girl Scout Cookies	40	Garden City 2	CO
Green Crack	65	Garden City 1	CO	Girl Scout Cookies	40	Garden City 3	CO
Green Crack	65	SLO 2	CA	Girl Scout Cookies	40	SLO 3	CA
Bruce Banner	60	Boulder 1	CO	Girl Scout Cookies	40	SLO 4	CA
Bruce Banner	60	Denver 1	CO	Girl Scout Cookies	40	Union Gap 1	WA
Bruce Banner	60	Denver 4	CO	Jack Flash	55	Boulder 1	CO
Bruce Banner	60	Fort Collins 3	CO	Jack Flash	55	Denver 3	CO
Bruce Banner	60	Fort Collins 4	CO	Larry OG	40	Boulder 1	CO
Bruce Banner	60	Garden City 1	CO	Larry OG	40	Denver 4	CO
Flo	60	Boulder 1	CO	Larry OG	40	SLO 3	CA
Flo	60	Denver 1	CO	G-13	30	Boulder 3	CO
Flo	60	Fort Collins 2	CO	G-13	30	Fort Collins 3	CO
Flo	60	Garden City 1	CO	G-13	30	Garden City 2	CO
Jillybean	60	Garden City 1	CO	Lemon Diesel	30	Boulder 1	CO
Jillybean	60	Garden City 2	CO	Lemon Diesel	30	Garden City 2	CO
Jillybean	60	Greeley 1	CO	Hash Plant	20	Fort Collins 3	CO
Pineapple Express	60	Boulder 1	CO	Hash Plant (Australian)	20	Garden City 1	CO
Pineapple Express	60	Denver 1	CO	Hash Plant	20	Garden City 1	CO
Pineapple Express	60	Garden City 2	CO	Hash Plant	20	Garden City 2	CO
Pineapple Express	60	Longmont 1	CO	Bubba Kush 98	20	Denver 1	CO
Pineapple Express	60	Union Gap	WA	Pre-98 Bubba Kush	15	Fort Collins 3	CO
Purple Haze	60	Denver 4	CO	Grape Ape	0	Boulder 1	CO
Purple Haze	60	Greeley 1	CO	Grape Ape	0	Union Gap 1	WA
Purple Haze	60	Fort Collins 1	CO	Purple Kush	0	Denver 1	CO
Tangerine^b^	60	Denver 1	CO	Purple Kush	0	Garden City 3	CO
Tangerine^b^	60	Garden City 1	CO	Purple Kush	0	Garden City 4	CO
Jack Herer	55	Garden City 3	CO				
Jack Herer	55	SLO 1	CA				
Jack Herer	55	Union Gap 1	WA				

^a^Strain proportion of “Chemdawg” variants not listed on Wikileaf

^b^Strain proportion of “Tangerine” not listed on Wikileaf; proportion listed is of “Tangerine Dream”

**Table 2:** Summary of *Cannabis* samples (122) from 30 strains with the reported proportion of Sativa retrieved from Wikileaf (2018). Abbreviations used for Lynch & Ritland (1999) relatedness statistics (Additional file 4: Figure S3) are included, and the proportions of membership for genotype 1 and genotype 2 from the STRUCTURE (Fig. 1) expressed as a percentage

Strain	Abbr	# Samples	Sativa Percentage	Genotype 1 (% average)	Genotype 2 (% average)	Standard Deviation
Durban Poison^a^	DuPo	9	100	86	14	9.9
Hawaiian	Hawa	2	90	61	39	27.58
Sour Diesel^a^	SoDi	7	90	14	86	53.74
Trainwreck	TrWr	2	90	59	41	21.92
Island Sweet Skunk	ISS	3	80	93	7	9.19
AK-47	AK47	3	65	55	45	7.07
Golden Goat^ab^	GoGo	7	65	68	32	2.12
Green Crack^b^	GrCr	3	65	60	40	3.54
Bruce Banner^a^	BrBa	6	60	19	81	28.99
Flo^a^	Flo	4	60	38	62	15.56
Jillybean	JiBe	3	60	73	27	9.19
Pineapple Express^a^	PiEx	5	60	62	38	1.41
Purple Haze	PuHa	3	60	77	23	12.02
Tangerine	Tang	2	60	53	47	4.95
Jack Herer	JaHe	3	55	66	34	7.78
OG Kush^ab^	OGKu	4	55	28	72	19.09
Blue Dream^ab^	BlDr	9	50	80	20	21.21
Tahoe OG	TaOG	4	50	26	74	16.97
Chemdawg^a^	ChDa	7	45	9	91	25.46
Headband	HeBa	2	45	57	43	8.49
Banana Kush^a^	BaKu	4	40	52	48	8.49
Girl Scout Cookies^ab^	GSC	8	40	25	75	10.61
Jack Flash	JaFl	2	40	96	4	39.6
Larry OG	LaOG	3	40	7	93	23.33
G-13	G13	3	30	50	50	14.14
Lemon Diesel^b^	LeDi	2	30	85	15	38.89
Hash Plant	HaPl	4	20	37	63	12.02
Pre98-Bubba Kush	PBK	2	15	7	93	5.66
Grape Ape	GrAp	2	0	55	45	38.89
Purple Kush^ab^	PuKu	4	0	29	71	20.51

^a^Twelve popular strains

^b^Clone only strains (SeedFinder 2018a)

### Microsatellite development

The *Cannabis* draft genome from “Purple Kush” (GenBank accession AGQN00000000.1) was scanned for microsatellite repeat regions using MSATCOMMANDER-1.0.8-beta (Faircloth 2008). Primers were developed *de-novo* flanking microsatellites with 3–6 nucleotide repeat units (Additional file 1: Table S2). Seven of the microsatellites had trinucleotide motifs, two had hexanucleotide motifs, and one had a tetranucleotide motif (Additional file 1: Table S2). One primer in each pair was tagged with a 5′ universal sequence (M13 or T7) so that a matching sequence with a fluorochrome tag could be incorporated via PCR (Schwabe et al. 2015). Ten primer pairs produced consistent peaks within the predicted size range and were used for the genetic analyses herein (Additional file 1: Table S2).

### PCR and data scoring

Microsatellite loci (Additional file 1: Table S2) were amplified in 12 μL reactions using 1.0 μL DNA (10–20 ng/ μL), 0.6 μL fluorescent tag (5 μM; FAM, VIC, or PET), 0.6 μL non-tagged primer (5 μM), 0.6 μL tagged primer (0.5 μM), 0.7 μL dNTP mix (2.5 mM), 2.4 μL GoTaq Flexi Buffer (Promega, Madison, WI, USA), 0.06 μL GoFlexi taq polymerase (Promega), 0.06 μL BSA (Bovine Serum Albumin 100X), 0.5–6.0 μL MgCl or MgSO_4_, and 0.48–4.98 μL dH_2_O. An initial 5 min denaturing step was followed by thirty five amplification cycles with a 1 min denaturing at 95 °C, 1 min annealing at primer-specific temperatures and 1 min extension at 72 °C. Two multiplexes (Additional file 1: Table S2) based on fragment size and fluorescent tag were assembled and 2 μL of each PCR product were combined into multiplexes up to a total volume of 10 μL. From the multiplexed product, 2 μL was added to Hi-Di formamide and LIZ 500 size standard (Applied Biosystems, Foster City, CA, USA) for electrophoresis on a 3730 Genetic Analyzer (Applied Biosystems) at the Arizona State University DNA Lab. Fragments were sized using GENEIOUS 8.1.8 (Biomatters Ltd).

### Genetic statistical analyses

GENALEX ver. 6.4.1 (Peakall and Smouse 2006; Peakall and Smouse 2012) was used to calculate deviation from Hardy–Weinberg equilibrium (HWE) and number of alleles for each locus (Additional file 1: Table S2). Linkage disequilibrium was tested using GENEPOP ver. 4.0.10 (Raymond and Rousset 1995; Rousset 2008). Presence of null alleles was assessed using MICRO-CHECKER (Van Oosterhout et al. 2004). Genotypes were analyzed using the Bayesian cluster analysis program STRUCTURE ver. 2.4.2 (Pritchard et al. 2000). Burn-in and run-lengths of 50,000 generations were used with ten independent replicates for each STRUCTURE analysis. STRUCTURE HARVESTER (Earl and vonHoldt 2012) was used to determine the *K* value to best describe the likely number of genetic groups for the data set. GENALEX produced a Principal Coordinate Analysis (PCoA) to examine variation in the dataset. Lynch & Ritland (1999) mean pairwise relatedness (*r*) statistics were calculated between all 122 samples resulting in 7381 pairwise *r*-values showing degrees of relatedness. For all strains the *r-*mean and standard deviation (SD) was calculated averaging among all samples. Obvious outliers were determined by calculating the lowest *r-*mean and iteratively removing those samples to determine the relatedness among the remaining samples in the subset. A graph was generated for 12 popular strains (Table 2) to show how the *r-*mean value change within a strain when outliers were removed.

## Results

The microsatellite analyses show genetic inconsistencies in *Cannabis* strains acquired from different facilities. While popular strains were widely available, some strains were found only at two dispensaries (Table 1). Since the aim of the research was not to identify specific locations where strain inconsistencies were found, dispensaries are coded to protect the identity of businesses.

There was no evidence of linkage-disequilibrium when all samples were treated as a single population. All loci deviate significantly from HWE, and all but one locus was monomorphic in at least two strains. All but one locus had excess homozygosity and therefore possibly null alleles. Given the inbred nature and extensive hybridization of *Cannabis*, deviations from neutral expectations are not surprising, and the lack of linkage-disequilibrium indicates that the markers are spanning multiple regions of the genome. The number of alleles ranged from 5 to 10 across the ten loci (Additional file 1: Table S2). There was no evidence of null alleles due to scoring errors.

**Fig. 1:**
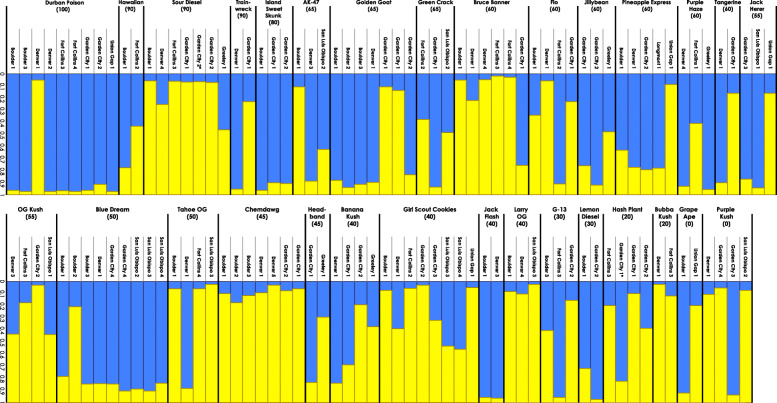
Bar plot graphs generated from STRUCTURE analysis for 122 individuals from 30 strains dividing genotypes into two genetic groups, K=2. Samples were arranged by purported proportions from 100% Sativa to 100% Indica (Wikileaf 2018) and then alphabetically within each strain by city. Each strain includes reported proportion of Sativa in parentheses (Wikileaf 2018) and each sample includes the coded location and city from where it was acquired. Each bar indicates proportion of assignment to genotype 1 (blue) and genotype 2 (yellow)

STRUCTURE HARVESTER calculated high support (∆K = 146.56) for two genetic groups, *K =* 2 (Additional file 2: Figure S1). STRUCTURE assignment is shown in Fig. 1 with the strains ordered by the purported proportions of Sativa phenotype (Wikileaf 2018). A clear genetic distinction between Sativa and Indica types would assign 100% Sativa strains (“Durban Poison”) to one genotype and assign 100% Indica strains (“Purple Kush”) to the other genotype (Table 2, Fig. 1, Additional file 3: Figure S2). Division into two genetic groups does not support the commonly described Sativa and Indica phenotypes. “Durban Poison” and “Purple Kush” follow what we would expect if there was support for the Sativa/Indica division. Seven of nine “Durban Poison” (100% Sativa) samples had 96% assignment to genotype 1, and three of four “Purple Kush” (100% Indica) had 89% assignment to genotype 2 (Fig. 1, Additional file 3: Figure S2). However, samples of “Hawaiian” (90% Sativa) and “Grape Ape” (100% Indica) do not show consistent patterns of predominant assignment to genotype 1 or 2. Interestingly, two predominantly Sativa strains “Durban Poison” (100% Sativa) and “Sour Diesel” (90% Sativa) have 86 and 14% average assignment to genotype 1, respectively. Hybrid strains such as “Blue Dream” and “Tahoe OG” (50% Sativa) should result in some proportion of shared ancestry, with assignment to both genotype 1 and 2. Eight of nine samples of “Blue Dream” show > 80% assignment to genotype 1, and three of four samples of “Tahoe OG” show < 7% assignment to genotype 1.

**Fig. 2:**
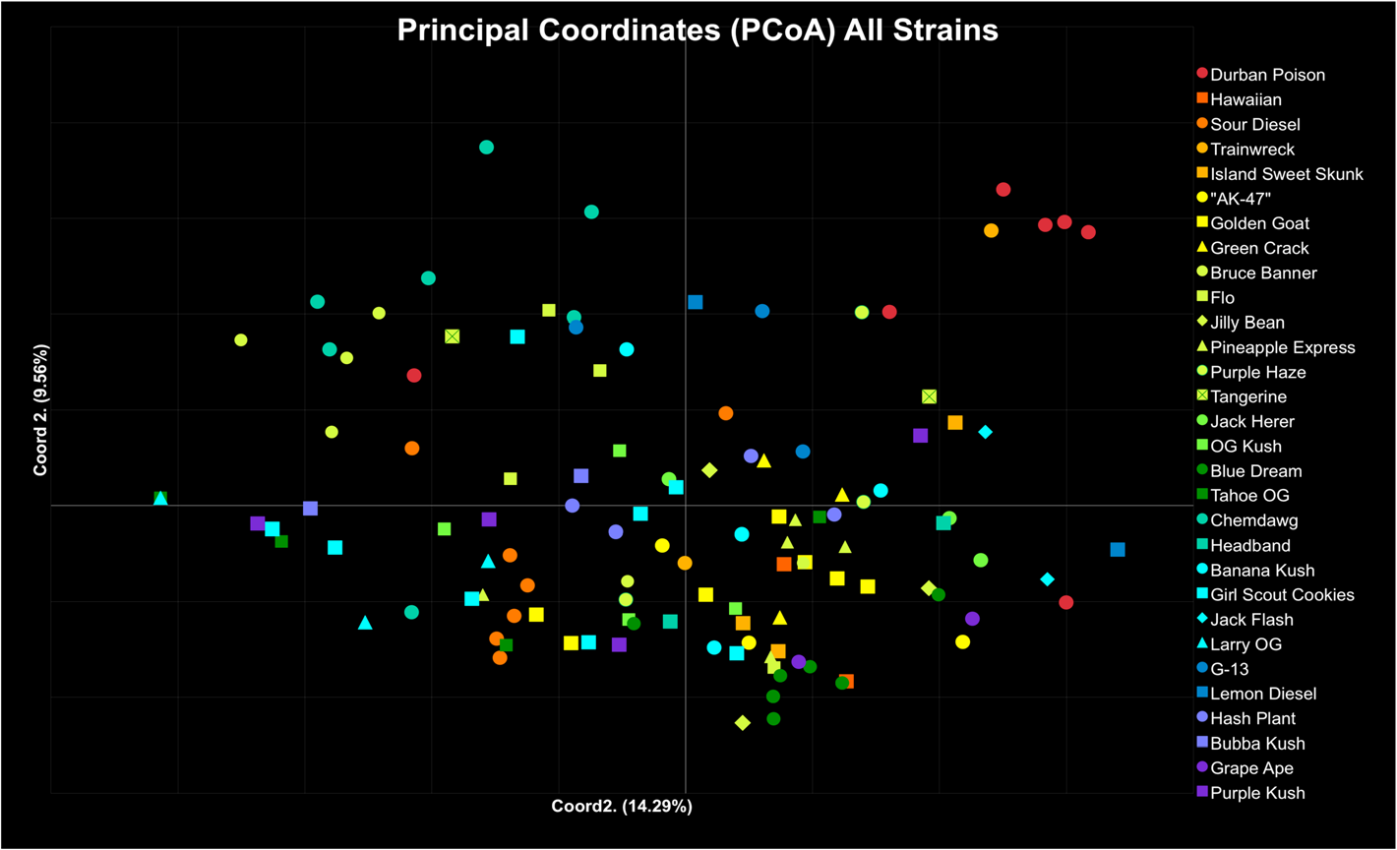
Principal Coordinates Analysis (PCoA) generated in GENALEX using Nei’s genetic distance matrix. Samples are a color-coded continuum by proportion of Sativa (Table 1) with the strain name given for each sample: Sativa type (red: 100% Sativa proportion, Hybrid type (dark green:50% Sativa proportion), and Indica type (purple: 0% Sativa proportion). Different symbols are used to indicate different strains within reported phenotype. Coordinate axis 1 explains 14.29% of the variation, coordinate axis 2 explains 9.56% of the variation, and Coordinate axis 3 (not shown) explains 7.07%

A Principal Coordinate Analyses (PCoA) was conducted using GENALEX (Fig. 2). Principal Coordinate Analyses (PCoA) is organized by color from 100% Sativa types (red), through all levels of Hybrid types (green 50:50), to 100% Indica types (purple; Fig. 2). Strain types with the same reported proportions are the same color but have different symbols. The PCoA of all strains represents 14.90% of the variation in the data on coordinate axis 1, 9.56% on axis 2, and 7.07% on axis 3 (not shown).

**Fig. 3:**
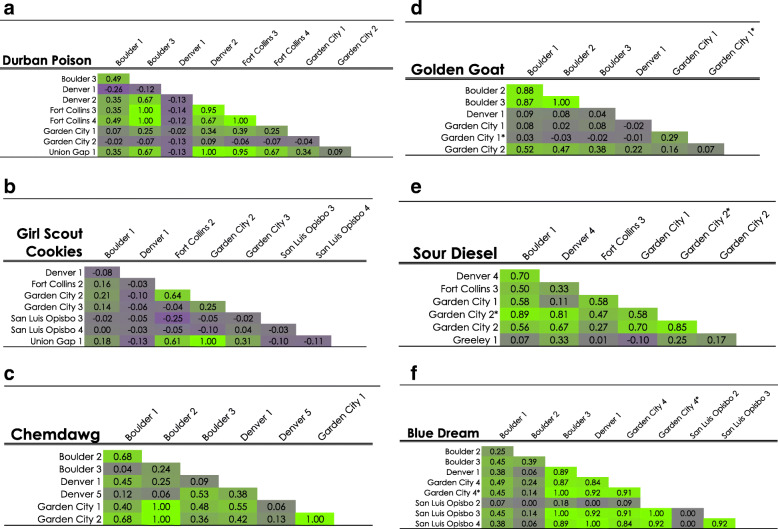
Heat maps of six prominent strains (**a**-**f**) using Lynch & Ritland (Faircloth 2008) pairwise genetic relatedness (*r*) values: purple indicates no genetic relatedness (minimum value -1.09) and green indicates a high degree of relatedness (maximum value 1.0). Sample strain names and location of origin are indicated along the top and down the left side of the chart. Pairwise genetic relatedness (*r*) values are given in each cell and cell color reflects the degree to which two individuals are related

Lynch & Ritland (1999) pairwise genetic relatedness (*r*) between all 122 samples was calculated in GENALEX. The resulting 7381 pairwise *r*-values were converted to a heat map using purple to indicate the lowest pairwise relatedness value (− 1.09) and green to indicate the highest pairwise relatedness value (1.00; Additional file 4: Figure S3). Comparisons are detailed for six popular strains (Fig. 3) to illustrate the relationship of samples from different sources and the impact of outliers. Values of close to 1.00 indicate a high degree of relatedness (Lynch and Ritland 1999), which could be indicative of clones or seeds from the same mother (Green 2005; SeedFinder 2018a). First order relatives (full siblings or mother-daughter) share 50% genetic identity (*r-*value = 0.50), second order relatives (half siblings or cousins) share 25% genetic identity (*r-*value = 0.25), and unrelated individuals are expected to have an *r-*value of 0.00 or lower. Negative values arise when individuals are less related than expected under normal panmictic conditions (Moura et al. 2013; Norman et al. 2017).

**Fig. 4:**
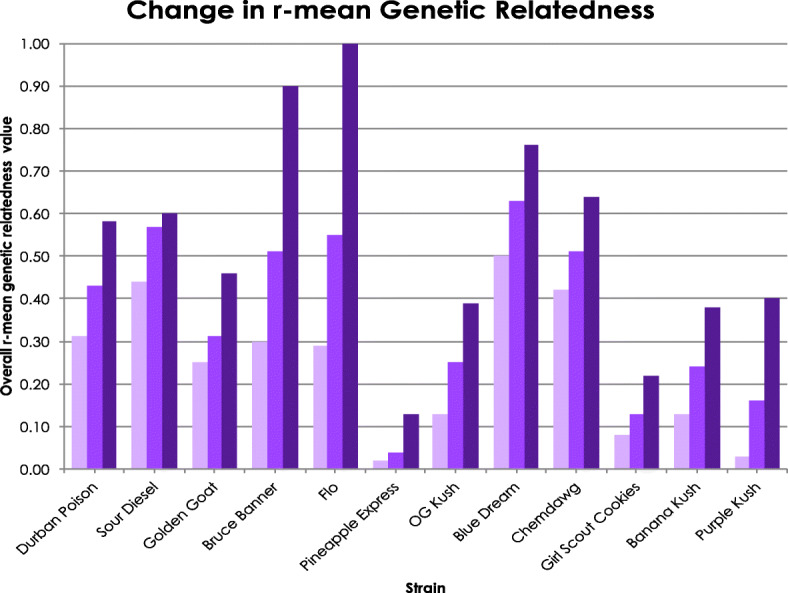
This graph indicates the mean pairwise genetic relatedness (*r*) initially (light purple), and after the removal of one (medium purple) or two (dark purple) outlying samples in 12 popular strains

Individual pairwise *r-*values were averaged within strains to calculate the overall *r-*mean as a measure of genetic similarity within strains which ranged from − 0.22 (“Tangerine”) to 0.68 (“Island Sweet Skunk”) (Table 3). Standard deviations ranged from 0.04 (“Jack Herer”) to 0.51 (“Bruce Banner”). The strains with higher standard deviation values indicate a wide range of genetic relatedness within a strain, while low values indicate that samples within a strain share similar levels of genetic relatedness. In order to determine how outliers impact the overall relatedness in a strain, the farthest outlier (lowest pairwise *r-*mean value) was removed and the overall *r-*means and SD values within strains were recalculated (Table 3). In all strains, the overall *r-*means increased when outliers were removed. In strains with more than three samples, a second outlier was removed and the overall *r-*means and SD values were recalculated. Overall *r-*means were used to determine degree of relatedness as clonal (or from stable seed; overall *r-*means > 0.9), first or higher order relatives (overall *r-*means 0.46–0.89), second order relatives (overall *r-*means 0.26–0.45), low levels of relatedness (overall *r-*means 0.00–0.25), and not related (overall *r-*means < 0.00). Overall *r-*means are displayed for all 30 strains (Table 3), and graphically for 12 popular strains (Fig. 4). Initial overall *r-*means indicate only three strains are first or higher order relatives (Table 3). Removing first or second outliers, depending on sample size, revealed that the remaining samples for an additional ten strains are first or higher order relatives (0.46–1.00), three strains are second order relatives (*r-*means 0.26–0.45), ten strains show low levels of relatedness (*r-*means 0.00–0.25; Table 3), and five strains are not related (*r-*means < 0.00). The impact of outliers can be clearly seen in the heat map for “Durban Poison” which shows the relatedness for 36 comparisons (Fig. 3a), six of which are nearly identical (*r*-value 0.90–1.0), while 13 are not related (*r*-value < 0.00). However, removal of two outliers, Denver 1 and Garden City 2, reduces the number of comparisons ranked as not related from 13 to zero.

**Table 3:** Lynch & Ritland (1999) pairwise relatedness comparisons of overall *r*-means (Mean) and standard deviations (SD) for samples of 30 strains including *r*-mean and SD after the first and second (where possible) outliers were removed. Outliers were samples with the lowest *r*-mean

Strain	# Samples	Measure	All samples	Outlier 1 removed	Outlier 2 removed
Durban Poison^a^	9	Mean	0.31	0.43	0.58
		SD	0.40	0.37	0.30
Hawaiian	2	Mean	−0.12	–	–
		SD			
Sour Diesel^a^	7	Mean	0.44	0.57	0.60
		SD	0.29	0.22	0.18
Trainwreck	2	Mean	−0.01	–	–
		SD			
Island Sweet Skunk	3	Mean	0.68	1.00	–
		SD	0.28		
AK-47	3	Mean	0.16	0.45	–
		SD	0.25		
Golden Goat^ab^	7	Mean	0.25	0.31	0.46
		SD	0.32	0.36	0.36
Green Crack^b^	3	Mean	0.38	0.89	–
		SD	0.46		
Bruce Banner^a^	6	Mean	0.30	0.51	0.90
		SD	0.51	0.50	0.05
Flo^a^	4	Mean	0.29	0.55	–
		SD	0.38	0.39	–
Jillybean	3	Mean	−0.03	0.04	–
		SD	0.12		
Pineapple Express^a^	5	Mean	0.02	0.04	0.13
		SD	0.16	0.17	0.19
Purple Haze	3	Mean	0.041	0.26	–
		SD	0.20		
Tangerine	2	Mean	−0.22	–	–
		SD			
Jack Herer	3	Mean	0.10	0.13	–
		SD	0.04		
OG Kush^ab^	4	Mean	0.13	0.25	–
		SD	0.19	0.22	–
Blue Dream^ab^	9	Mean	0.50	0.63	0.76
		SD	0.39	0.34	0.24
Tahoe OG	4	Mean	0.21	0.41	0.54
		SD	0.26		
Chemdawg^a^	7	Mean	0.42	0.51	0.64
		SD	0.31	0.31	0.28
Headband	2	Mean	0.11	–	–
		SD			
Banana Kush^a^	4	Mean	0.13	0.24	–
		SD	0.20	0.13	–
Girl Scout Cookies^ab^	8	Mean	0.08	0.13	0.22
		SD	0.27	0.30	0.32
Jack Flash	2	Mean	0.62	–	–
		SD			
Larry OG	3	Mean	0.32	0.67	–
		SD	0.31		
G-13	3	Mean	0.29	0.56	–
		SD	0.32		
Lemon Diesel^b^	2	Mean	0.10	–	–
		SD			
Hash Plant	4	Mean	0.25	0.25	0.43
		SD	0.27	0.17	
Pre98-Bubba Kush	2	Mean	−0.02	–	–
		SD			
Grape Ape	2	Mean	−0.05	–	–
		SD			
Purple Kush^ab^	4	Mean	0.03	0.16	–
		SD	0.21	0.22	–

^a^Twelve popular strains

^b^Clone only strains (SeedFinder 2018a)

## Discussion

*Cannabis* is becoming an ever-increasing topic of discussion, so it is important that scientists and the public can discuss *Cannabis* in a similar manner. Currently, not only are Sativa and Indica types disputed (Emboden 1974; Hillig 2005; Russo 2007; Clarke and Merlin 2013; Clarke et al. 2015; Clarke and Merlin 2016; McPartland 2017; Piomelli and Russo 2016; Small 2015b; De Meijer and Keizer 1996), but experts also are at odds about nomenclature for *Cannabis* (Emboden 1974; Hillig 2005; Russo 2007; Clarke and Merlin 2013; Clarke et al. 2015; Clarke and Merlin 2016; McPartland 2017; Piomelli and Russo 2016; Small 2015b; De Meijer and Keizer 1996). We postulated that genetic profiles from samples with the same strain identifying name should have identical, or at least, highly similar genotypes no matter the source of origin. The multiple genetic analyses used here address paramount questions for the medical *Cannabis* community and bring empirical evidence to support claims that inconsistent products are being distributed. An important element for this study is that samples were acquired from multiple locations to maximize the potential for variation among samples. Maintenance of the genetic integrity through genotyping is possible only following evaluation of genetic consistency and continuing to overlook this aspect will promote genetic variability and phenotypic variation within *Cannabis*. Addressing strain variability at the molecular level is of the utmost importance while the industry is still relatively new.

Genetic analyses have consistently found genetic distinction between hemp and marijuana, but no clear distinction has been shown between the common description of Sativa and Indica types (Lynch et al. 2016; Soler et al. 2017; Sawler et al. 2015; Dufresnes et al. 2017; De Meijer and Keizer 1996). We found high support for two genetic groups in the data (Fig. 1) but no discernable distinction or pattern between the described Sativa and Indica strains. The color-coding of strains in the PCoA for all 122 samples allows for visualization of clustering among similar phenotypes by color: Sativa (red/orange), Indica (blue/purple) and Hybrid (green) type strains (Fig. 2). If genetic differentiation of the commonly perceived Sativa and Indica types previously existed, it is no longer detectable in the neutral genetic markers used here. Extensive hybridization and selection have presumably created a homogenizing effect and erased evidence of potentially divergent historical genotypes.

Wikileaf maintains that the proportions of Sativa and Indica reported for strains are largely based on genetics and lineage (Nelson 2016), although online databases do not give scientific evidence for their categorization other than parentage information from breeders and expert opinions. This has seemingly become convoluted over time (Russo 2007; Clarke and Merlin 2013; Small 2015a; Small 2016). Our results show that commonly reported levels of Sativa, Indica and Hybrid type strains are often not reflected in the average genotype. For example, two described Sativa type strains “Durban Poison” and “Sour Diesel”, have contradicting genetic assignments (Fig. 1, Table 2). This analysis indicates strains with similar reported proportions of Sativa or Indica may have differing genetic assignments. Further illustrating this point is that “Bruce Banner”, “Flo”, “Jillybean”, “Pineapple Express”, “Purple Haze”, and “Tangerine” are all reported to be 60/40 Hybrid type strains, but they clearly have differing levels of admixture both within and among these reportedly similar strains (Table 2, Fig. 1). From these results, we can conclude that reported ratios or differences between Sativa and Indica phenotypes are not discernable using these genetic markers. Given the lack of genetic distinction between Indica and Sativa types, it is not surprising that reported ancestry proportions are also not supported.

To accurately address reported variation within strains, samples were purchased from various locations, as a customer, with no information of strains other than publicly available online information. Evidence for genetic inconsistencies is apparent within many strains and supported by multiple genetic analyses. Soler et al. (2017) found genetic variability among seeds from the same strain supplied from a single source, indicating genotypes within strains are variable. When examining the STRUCTURE genotype assignments, it is clear that many strains contained one or more divergent samples with a difference of > 0.10 genotype assignment (e.g. “Durban Poison” – Denver 1; Figs. 1, 3a). Of the 30 strains examined, only four strains had consistent STRUCTURE genotype assignment and admixture among all samples. The number of strains with consistent STRUCTURE assignments increased to 11 and 15 when one or two samples were ignored, respectively. These results indicate that half of the included strains showed relatively stable genetic identity among most samples. Six strains had only two samples, both of which were different (e.g., “Trainwreck” and “Headband”). The remaining nine strains in the analysis had more than one divergent sample (e.g., “Sour Diesel”) or had no consistent genetic pattern among the samples within the strain (e.g., “Girl Scout Cookies”; Table 3, Figs. 1, 2, Additional file 3: Figure S2). It is noteworthy that many of the strains used here fell into a range of genetic relatedness indicative of first order siblings (*see Lynch & Ritland analysis below*) when samples with high genetic divergence were removed from the data set (Table 3; Figs. 3, 4). Eight of the 30 strains examined are identified as clone only (Table 2). All eight of the strains described as clone only show differentiation of at least one sample within the strain (Fig. 1). For example, one sample of “Blue Dream” is clearly differentiated from the remaining eight, and “Girl Scout Cookies” has little genetic cohesiveness among the eight samples (Figs. 1, 2). Other genetic studies have similarly found genetic inconsistencies across samples within the same strain (Lynch et al. 2016; Soler et al. 2017; Sawler et al. 2015). These results lend support to the idea that unstable genetic lines are being used to produce seed.

A pairwise genetic heat map based on Lynch & Ritland (1999) pairwise genetic relatedness (*r-*values) was generated to visualize genetic relatedness throughout the data set (Additional file 4: Figure S3). Values of 1.00 (or close to) are assumed to be clones or plants from self-fertilized seed. Six examples of within-strain pairwise comparison heat maps were examined to illustrate common patterns (Fig. 3). The heat map shows that many strains contain samples that are first order relatives or higher (*r*-value > 0.49). For example, “Sour Diesel” (Fig. 3) has 12 comparisons of first order or above, and six have low/no relationship. There are also values that could be indicative of clones or plants from a stable seed source such as “Blue Dream” (Fig. 3), which has 10 nearly identical comparisons (*r*-value 0.90–1.00), and no comparisons in “Blue Dream” have negative values. While “Blue Dream” has an initial overall *r*-mean indicating first order relatedness within the samples (Table 3, Fig. 4), it still contains more variation than would be expected from a clone only strain (Clone Only Strains n.d.). Other clone-only strains (Clone Only Strains n.d.) e.g. “Girl Scout Cookies” (Table 3, Fig. 3) and “Golden Goat” (Table 3, Fig. 3), have a high degree of genetic variation resulting in low overall relatedness values. Outliers were calculated and removed iteratively to demonstrate how they affected the overall *r*- mean within the 12 popular strains (Table 3, Fig. 4). In all cases, removing outliers increased the mean *r*-value, as illustrated by “Bruce Banner”, which increased substantially, from 0.3 to 0.9 when samples with two outlying genotypes were removed. There are unexpected areas in the entire dataset heat map that indicate high degrees of relatedness between different strains (Additional file 4: Figure S3). For example, comparisons between “Golden Goat” and “Island Sweet Skunk” (overall *r*- mean 0.37) are higher than within samples of “Sour Diesel”. Interestingly, “Golden Goat” is reported to be a hybrid descendant of “Island Sweet Skunk” (Leafly 2018a; Wikileaf 2018; NCSM 2018; PotGuide.com 2018; Seedfinder 2018) which could explain the high genetic relatedness between these strains. However, most of the between strain overall *r*- mean are negative (e.g., “Golden Goat” to “Durban Poison” -0.03 and “Chemdawg” to “Durban Poison” -0.22; Additional file 4: Figure S3), indicative of limited recent genetic relationship.

While collecting samples from various dispensaries, it was noted that strains of “Chemdawg” had various different spellings of the strain name, as well as numbers and/or letters attached to the name. Without knowledge of the history of “Chemdawg”, the assumption was that these were local variations. These were acquired to include in the study to determine if and how these variants were related. Upon investigation of possible origins of “Chemdawg”, an interesting history was uncovered, especially in light of the results. Legend has it that someone named “Chemdog” (a person) grew the variations (“Chem Dog”, “Chem Dog D”, “Chem Dog 4”) from seeds he found in a single bag of *Cannabis* purchased at a Grateful Dead concert (Danko 2016). However, sampling suggests dispensaries use variations of the name, and more often the “Chemdawg” form of the name is used, albeit incorrectly (Danko 2016). The STRUCTURE analysis indicates only one “Chemdawg” individual has > 0.10 genetic divergence compared to the other six samples (Fig. 1, Additional file 3: Figure S2). Five of seven “Chemdawg” samples cluster in the PCoA (Fig. 2), and six of seven “Chemdawg” samples are first order relatives (*r*-value > 0.50; Table 3, Fig. 3). The history of “Chem Dog” is currently unverifiable, but the analysis supports that these variations could be from seeds of the same plant. This illustrates how *Cannabis* strains may have come to market in a non-traditional manner. Genetic analyses can add scientific support to the stories behind vintage strains and possibly help clarify the history of specific strains.

Genetic inconsistencies may come from both suppliers and growers of *Cannabis* clones and stable seed, because currently they can only assume the strains they possess are true to name. There is a chain of events from seed to sale that relies heavily on the supplier, grower, and dispensary to provide the correct product, but there is currently no reliable way to verify *Cannabis* strains. The possibility exists for errors in plant labeling, misplacement, misspelling (e.g. “Chem Dog” vs. “Chemdawg”), and/or relabeling along the entire chain of production. Although the expectation is that plants are labeled carefully and not re-labeled with a more desirable name for a quick sale, these misgivings must be considered. Identification by genetic markers has largely eliminated these types of mistakes in other widely cultivated crops such as grapes, olives and apples. Modern genetic applications can accurately identify varieties and can clarify ambiguity in closely related and hybrid species (Guilford et al. 1997; Hokanson et al. 1998; Sarri et al. 2006; Costantini et al. 2005; United States Department of Agriculture 2014).

Matching genotypes within the same strains were expected, but highly similar genotypes between samples of different strains could be the result of mislabeling or misidentification, especially when acquired from the same source. The pairwise genetic relatedness *r-*values were examined for incidence of possible mislabeling or re-labeling. There were instances in which different strains had *r*-values = 1.0 (Additional file 4: Figure S3), indicating clonal genetic relationships. Two samples with matching genotypes were obtained from the same location (“Larry OG” and “Tahoe OG” from San Luis Obispo 3). This could be evidence for mislabeling or misidentification because these two samples have similar names. It is unlikely that these samples from reportedly different strains have identical genotypes, and more likely that these samples were mislabeled at some point. Misspelling may also be a source of error, especially when facilities are handwriting labels. An example of possible misspelling may have occurred in the sample labeled “Chemdog 1” from Garden City 1. “Chemdawg 1”, a described strain, could have easily been misspelled, but it is unclear whether this instance is evidence for mislabeling or renaming a local variant. Inadvertent mistakes may carry through to scientific investigation where strains are spelled or labeled incorrectly. For example, Vergara et al. (2016) reports genome assemblies for “Chemdog” and “Chemdog 91” as they are reported in GenBank (GCA_001509995.1), but neither of these labels are recognized strain names. “Chemdawg” and “Chemdawg 91” are recognized strains (Leafly 2018a; Wikileaf 2018; cannabis.info 2018; NCSM 2018; PotGuide.com 2018; Seedfinder 2018), but according to the original source, the strain name “Chemdawg” is incorrect, and it should be “Chem Dog” (Danko 2016), but the name has clearly evolved among growers since it emerged in 1991 (Danko 2016). Another example that may lead to confusion is how information is reported in public databases. For example, data is available for the reported monoisolate of “Pineapple Banana Bubba Kush” in GenBank (SAMN06546749), and while “Pineapple Kush”, “Banana Kush” and “Bubba Kush” are known strains (Leafly 2018a; Wikileaf 2018; cannabis.info 2018; NCSM 2018; PotGuide.com 2018; Seedfinder 2018), the only record we found of “Pineapple Banana Bubba Kush” is in GenBank. This study has highlighted several possible sources of error and how genotyping can serve to uncover sources of variation. Although this study was unable to confirm sources of error, it is important that producers, growers and consumers are aware that there are errors and they should be documented and corrected whenever possible.

## Conclusions

Over the last decade, the legal status of *Cannabis* has shifted and is now legal for medical and some recreational adult use, in the majority of the United States as well as several other countries that have legalized or decriminalized *Cannabis.* The recent legal changes have led to an unprecedented increase in the number of strains available to consumers. There are currently no baseline genotypes for any strains, but steps should be taken to ensure products marketed as a particular strain are genetically congruent. Although the sampling in this study was not exhaustive, the results are clear: strain inconsistency is evident and is not limited to a single source, but rather exists among dispensaries across cities in multiple states. Various suggestions for naming the genetic variants do not seem to align with the current widespread definitions of Sativa, Indica, Hybrid, and Hemp (Hillig 2005; Clarke and Merlin 2013). As our *Cannabis* knowledge base grows, so does the communication gap between scientific researchers and the public. Currently, there is no way for *Cannabis* suppliers, growers or consumers to definitively verify strains. Exclusion from USDA protections due to the Federal status of *Cannabis* as a Schedule I drug has created avenues for error and inconsistencies. Presumably, the genetic inconsistencies will often manifest as differences in overall effects (Minkin 2014). Differences in characteristics within a named strain may be surprising for a recreational user, but differences may be more serious for a medical patient who relies on a particular strain for alleviation of specific symptoms.

This study shows that in neutral genetic markers, there is no consistent genetic differentiation between the widely held perceptions of Sativa and Indica *Cannabis* types. Moreover, the genetic analyses do not support the reported proportions of Sativa and Indica within each strain, which is expected given the lack of genetic distinction between Sativa and Indica. There may be land race strains that phenotypically and genetically separate as Sativa and Indica types, however our sampling does not include an adequate number of these strains to define these as two potentially distinct genotypes. The recent and intense breeding efforts to create novel strains has likely merged the two types and blurred previous separation between the two types. However, categorizing strains this way helps consumers communicate their preference for a spectrum of effects (e.g.: Sativa-dominant Hybrid), and the vernacular usage will likely continue to be used, despite a lack of evidence of genetic differentiation.

Instances we found where samples within strains are not genetically similar, which is unexpected given the manner in which *Cannabis* plants are propagated. Although it is impossible to determine the source of these inconsistencies as they can arise at multiple points throughout the chain of events from seed to sale, we theorize misidentification, mislabeling, misplacement, misspelling, and/or relabeling are all possible. Especially where names are similar, there is the possibility for mislabeling, as was shown here. In many cases genetic inconsistencies within strains were limited to one or two samples. We feel that there is a reasonable amount of genetic similarity within many strains, but currently there is no way to verify the “true” genotype of any strain. Although the sampling here includes merely a fragment of the available *Cannabis* strains, our results give scientific merit to previously anecdotal claims that strains can be unpredictable.

## Additional files

Additional file 1: **Table S1.** Twelve popular strains and their described assignment of Sativa and Indica according to six online data bases of Cannabis strain information. **Table S2.** Primer information includes the multiplex assignment, primer name, microsatellite repeat and number of units repeated in the “Purple Kush” draft genome (National Center for Biotechnology Information, accession AGQN00000000.1), forward and reverse sequences (asterisk denotes the sequence to which the tag is attached), the universal tag (sequence revealed at the bottom of the table), dye (VIC, FAM, PET), optimized annealing temperature, MgCl uL volume,amplified fragment size range, and the number of alleles in the data set. (XLSX 52 kb)

Additional file 2: **Figure S1.** STRUCTURE HARVESTER graph indicating K = 2 is highly supported. (ΔK = 146.56) as the number of genetic groups for this data. (PDF 55 kb)

Additional file 3: **Figure S2.** Bar plot graphs generated from STRUCTURE analysis for individuals from twelve popular strains (Table 2), dividing genotypes into two genetic groups, K = 2. Each sample includes the coded location and city from where it was acquired. Each bar indicates proportion of assignment to genotype 1 (blue) and genotype 2 (yellow). (PDF 65 kb)

Additional file 4: **Figure S3.** A genetic heat map chart of Lynch & Ritland pairwise genetic relatedness (*r*) values for 122 samples where purple indicates no genetic relatedness (minimum value − 1.09) and green indicates a high degree of relatedness (maximum value 1.0). Sample strain names and location of origin are indicated along the top and down the left side of the chart. Pairwise genetic relatedness (*r*) values are given in each cell and cell color reflects the degree to which two individuals are related. (PDF 239 kb)

## Abbreviations

CTAB: Cetyl trimethylammonium bromide
DNA: Deoxyribonucleic acid
HWE: Hardy–Weinberg equilibrium
PCoA: Principle Coordinates Analysis
PCR: Polymerase chain reaction
PTSD: Post-traumatic stress disorder
SD: Standard Deviation
SLO: San Luis Obispo
THC: Δ^9^-tetrahydrocannabinol
U.S.: United States
USDA: United States Department of Agriculture
