# Supplementary material for: Genetic tools weed out misconceptions of strain reliability in Cannabis sativa: implications for a budding industry
Source: J Cannabis Res. 2019 Jun 7;1:3. doi: 10.1186/s42238-019-0001-1 (PMC7815053; doi:10.1186/s42238-019-0001-1)
Supplement: Supplementary file 2 — Figure S1. STRUCTURE HARVESTER graph indicating K = 2 is highly supported. (ΔK = 146.56) as the number of genetic groups for this data. (PDF 55 kb) [file 42238_2019_1_MOESM2_ESM.pdf]

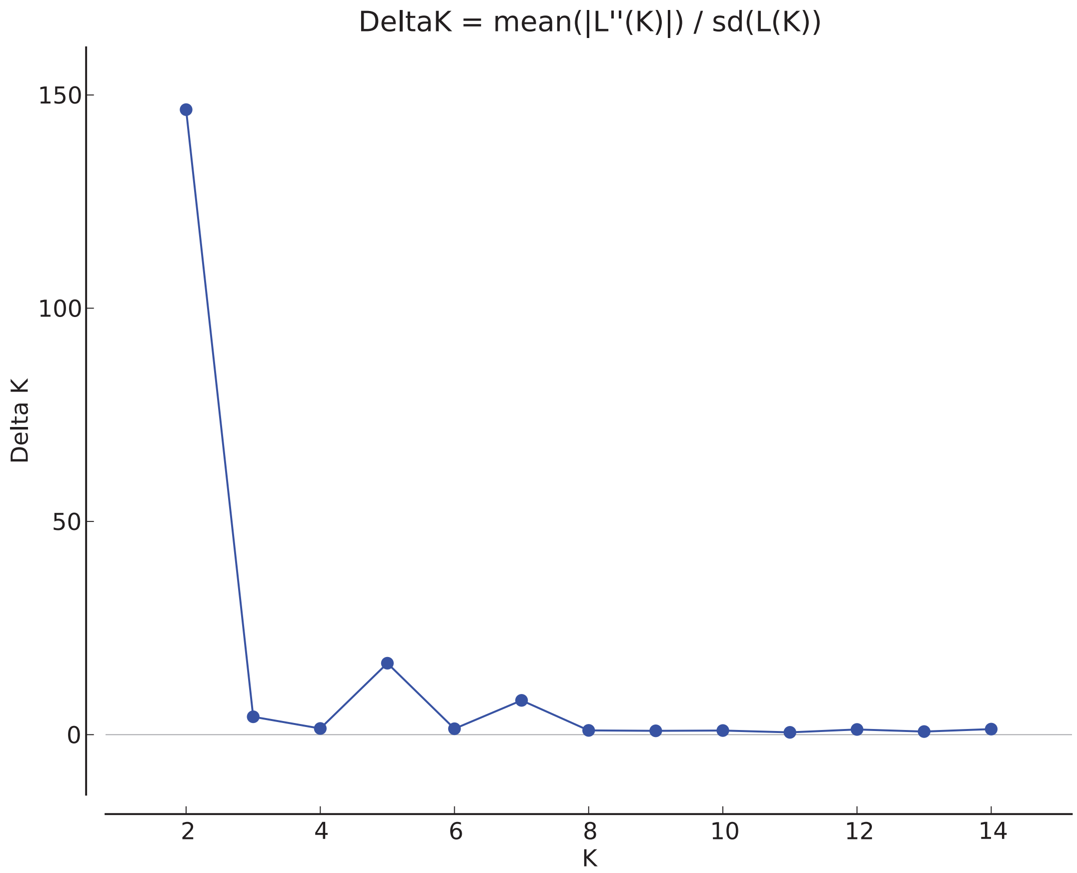

Figure S1.
STRUCTURE HARVESTER graph indicating K=2 is highly supported (ΔK=146.56) as the number of genetic groups for this data.
