# Supplementary material for: Genetic tools weed out misconceptions of strain reliability in Cannabis sativa: implications for a budding industry
Source: J Cannabis Res. 2019 Jun 7;1:3. doi: 10.1186/s42238-019-0001-1 (PMC7815053; doi:10.1186/s42238-019-0001-1)
Supplement: Supplementary file 3 — Figure S2. Bar plot graphs generated from STRUCTURE analysis for individuals from twelve popular strains (Table 2), dividing genotypes into two genetic groups, K = 2. Each sample includes the coded location and city from where it was acquired. Each bar indicates proportion of assignment to genotype 1 (blue) and genotype 2 (yellow). (PDF 65 kb) [file 42238_2019_1_MOESM3_ESM.pdf]

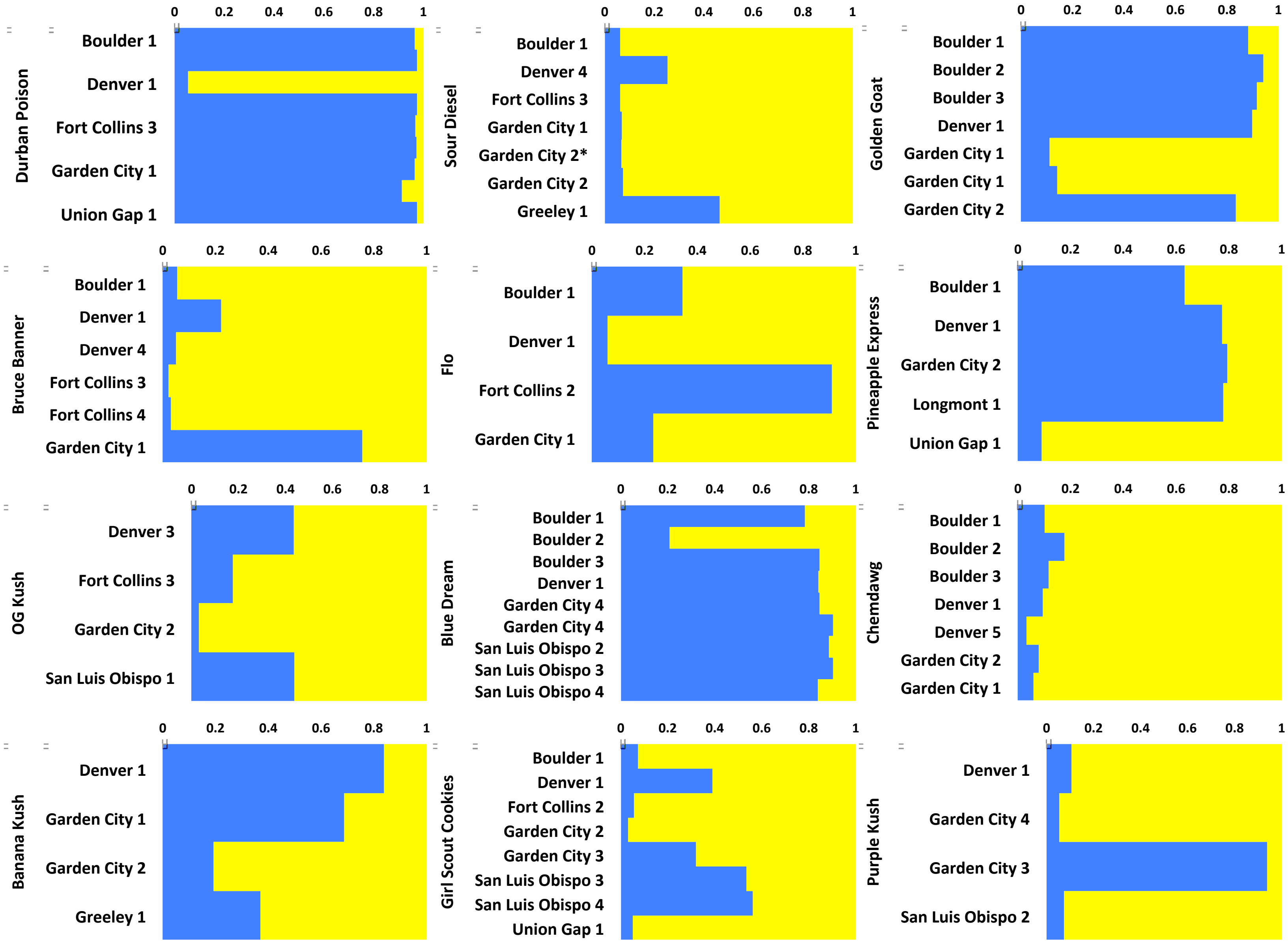

Figure S2.

Bar plot graphs generated from STRUCTURE analysis for individuals from twelve popular strains (Table 2), dividing genotypes into two genetic groups, K=2. Each sample includes the coded location and city from where it was acquired. Each bar indicates proportion of assignment to genotype 1 (blue) and genotype 2 (yellow)
