# Supplementary material for: Genetic tools weed out misconceptions of strain reliability in Cannabis sativa: implications for a budding industry
Source: J Cannabis Res. 2019 Jun 7;1:3. doi: 10.1186/s42238-019-0001-1 (PMC7815053; doi:10.1186/s42238-019-0001-1)
Supplement: Supplementary file 4 — Figure S3. A genetic heat map chart of Lynch & Ritland pairwise genetic relatedness (r) values for 122 samples where purple indicates no genetic relatedness (minimum value − 1.09) and green indicates a high degree of relatedness (maximum value 1.0). Sample strain names and location of origin are indicated along the top and down the left side of the chart. Pairwise genetic relatedness (r) values are given in each cell and cell color reflects the degree to which two individuals are related. (PDF 239 kb) [file 42238_2019_1_MOESM4_ESM.pdf]

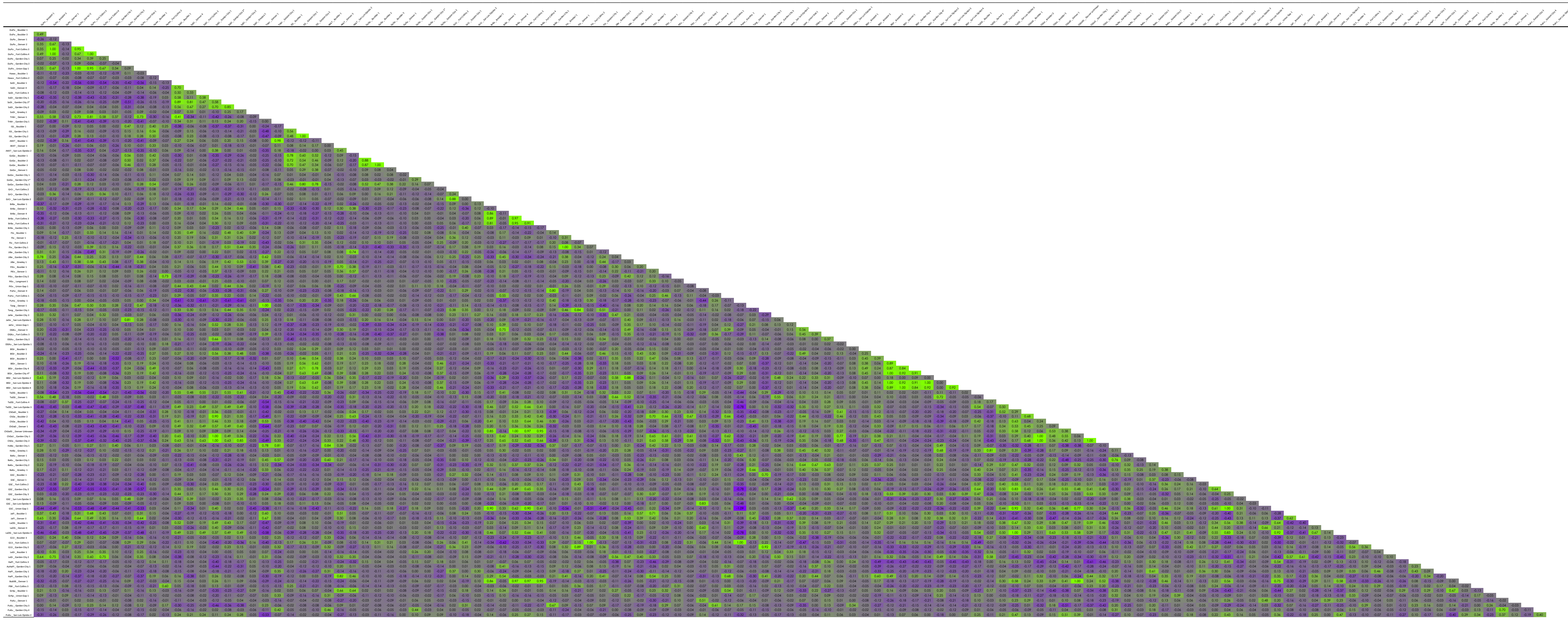

Figure S3.

A genetic heat map chart of Lynch & Ritland pairwise genetic relatedness (*r*) values for 122 samples where purple indicates no genetic relatedness (minimum value -1.09) and green indicates a high degree of relatedness (maximum value 1.0). Sample strain names and location of origin are indicated along the top and down the left side of the chart. Pairwise genetic relatedness (*r*) values are given in each cell and cell color reflects the degree to which two individuals are related.
